# TRANSFoRm eCRF

**DOI:** 10.1186/2043-9113-5-S1-S17

**Published:** 2015-05-22

**Authors:** Wolfgang Kuchinke, Theodoros N Arvanitis

**Affiliations:** 1 Heinrich-Heine University Düsseldorf, University Hospital, 40225 Düsseldorf, Germany; 2 University of Warwick, Coventry, CV4 7AL, UK

## Characterisation

Tool, data collection for clinical trials, open source, data management.

## Description

Data derived from electronic health records (EHRs) can be used by providing protocol developers with information about the feasibility of inclusion and exclusion criteria, by simplifying patient recruitment and improving the clinical trial data collection process through pre-filling electronic Case Report Forms (eCRF), thus avoiding double data collection for clinical care and research. This process is called secondary use of clinical data, and it can play a central role in a system that learns from data collected at the point of care and applies the lessons learned to the improvement of patient care.

As part of the TRANSFoRm platform, an eCRF [1] was built that interacts with the EHR at the physician’s practice to support data collection in clinical trials. It is employed in a clinical trial involving the identification of prevalent and incident cases of Gastro-Oesophageal Reflux Disease (GORD), a disease that occurs when stomach acid leaks up into the oesophagus. Patients are randomized to on-demand or continuous consumption of proton pump inhibitors (PPI). Data collection is done by using mobile health applications filled out by patients, and the eCRF completed by medically qualified personnel during practice visits. The distinctive feature of the TRANSFoRm eCRF is its integration with the EHR at the physician/investigator site and allows for the collection of semantically controlled data from within the EHR system. In addition, it provides CDISC ODM as representation format and supports semantic interoperability with clinical data sources.

**Figure 1:**
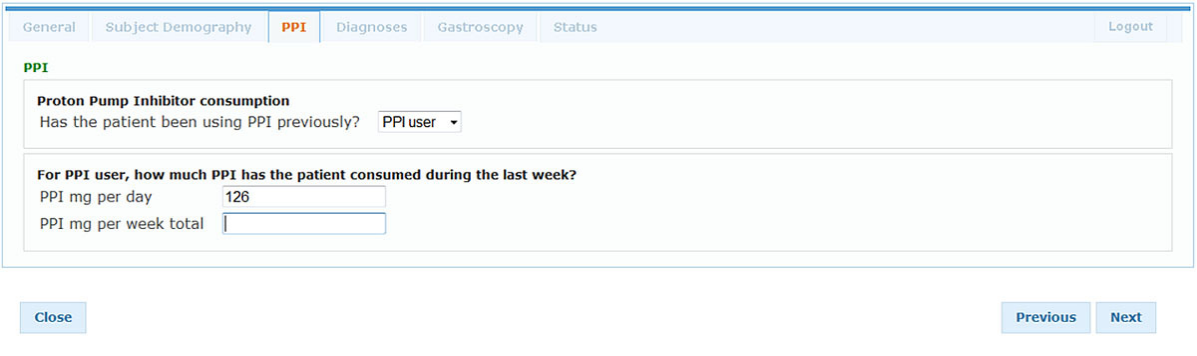
eCRF user interface for the collection of PPI consumption data.

During the initiation visit for the GORD trial and at month 3, 6, 9 and 12, information about PPI consumption is collected (Figure 1). In addition, event driven data collection from EHR/eCRF is triggered, for example, in the event of an adverse effect (AE). During the first visit of the patient, the eCRF is pre-populated with relevant data available in the EHR at that time. After confirmation by the GP/investigator that all eligibility criteria are met, a suitable eCRF form is automatically generated to record the enrollment of the patient into the trial and the results of randomization.

## Status of development

Is employed in the TRANSFoRm GORD study, and being validated for Good Clinical Practice (GCP). It will be provided as open source.

## Users

Physicians/investigators interested in using primary care data to improve clinical research.

## Link

http://www.transformproject.eu/
